# Rumen microbial and fermentation characteristics are affected differently by bacterial probiotic supplementation during induced lactic and subacute acidosis in sheep

**DOI:** 10.1186/1471-2180-12-142

**Published:** 2012-07-19

**Authors:** Abderzak Lettat, Pierre Nozière, Mathieu Silberberg, Diego P Morgavi, Claudette Berger, Cécile Martin

**Affiliations:** 1 INRA, UR1213 Herbivores, Centre de Recherches de Clermont-Ferrand/Theix, F-63122 Saint Genès, Champanelle, France; 2 Danisco France SAS, Zone d'Activités de Buxières, BP 10, F-86220, Dangé-Saint-Romain, France; 3 Danisco France SAS, 20 rue Brunel, F-75017 Paris, France; 4 Present address: Dairy and Swine R&D Centre, Sherbrooke, QC, Canada

**Keywords:** Acidosis, DGGE, Microbiota, Probiotics, qPCR, Rumen, SARA

## Abstract

**Background:**

Ruminal disbiosis induced by feeding is the cause of ruminal acidosis, a digestive disorder prevalent in high-producing ruminants. Because probiotic microorganisms can modulate the gastrointestinal microbiota, propionibacteria- and lactobacilli-based probiotics were tested for their effectiveness in preventing different forms of acidosis.

**Results:**

Lactic acidosis, butyric and propionic subacute ruminal acidosis (SARA) were induced by feed chalenges in three groups of four wethers intraruminally dosed with wheat, corn or beet pulp. In each group, wethers were either not supplemented (C) or supplemented with *Propionibacterium* P63 alone (P) or combined with *L. plantarum* (Lp + P) or *L. rhamnosus* (Lr + P). Compared with C, all the probiotics stimulated lactobacilli proliferation, which reached up to 25% of total bacteria during wheat-induced lactic acidosis. This induced a large increase in lactate concentration, which decreased ruminal pH. During the corn-induced butyric SARA, Lp + P decreased *Prevotella* spp*.* proportion with a concomitant decrease in microbial amylase activity and total volatile fatty acids concentration, and an increase in xylanase activity and pH. Relative to the beet pulp-induced propionic SARA, P and Lr + P improved ruminal pH without affecting the microbial or fermentation characteristics. Regardless of acidosis type, denaturing gradient gel electrophoresis revealed that probiotic supplementations modified the bacterial community structure.

**Conclusion:**

This work showed that the effectiveness of the bacterial probiotics tested depended on the acidosis type. Although these probiotics were ineffective in lactic acidosis because of a deeply disturbed rumen microbiota, some of the probiotics tested may be useful to minimize the occurrence of butyric and propionic SARA in sheep. However, their modes of action need to be further investigated.

## Background

The rumen constitutes an effective animal-microbe mutualism system from which both partners derive benefit [1]. Current feeding practices in high-producing beef and dairy cattle use highly fermentable diets to increase growth rates and milk production, but because of microbial disturbances, they predispose cattle to digestive disorders such as ruminal acidosis [2]. Field studies in Europe and the USA estimate that 11 to 19% of early lactation and 18 to 26% of mid-lactation dairy cows have subacute ruminal acidosis (SARA) [3]. As it affects animal health and reduces performance, SARA is considered to be the most important nutritional disorder for ruminants [4,5]. Among the strategies developed to prevent SARA, the use of chemical buffers [6], ionophores [7] and probiotics based on yeast such as *Saccharomyces cerevisiae*[8,9] have been found to stabilize ruminal pH and improve animal production. Contrastingly, there is less information on the use of bacterial probiotics. Supplementation with lactate-producing bacteria or combining them with bacteria that utilize lactate was reported to decrease lactate and increase propionate in the rumen and thus could help to prevent SARA [10,11]. However, positive effects of bacterial probiotics on ruminal pH were observed only when these were associated with yeast [11,12], and their effect on the ruminal microbiota has not yet received enough attention. Because several factors including animal models, diets, microbial strains and doses may affect probiotic effectiveness in preventing SARA, we hypothesized that the ruminal fermentation patterns could influence the effect of bacterial probiotics. In the present work, the effects of *Lactobacillus* and *Propionibacterium* supplementation on ruminal microbial and fermentation characteristics were investigated using a previously developed model of ruminal acidosis in wethers favoring lactic, propionic or butyric fermentation pathways [13].

## Methods

### Ethics statement

The experiment was conducted at the animal experimental facilities of the INRA Herbivores Research Unit (Saint-Genès Champanelle, France). Procedures on animals complied with the guidelines for animal research of the French Ministry of Agriculture and all other applicable national and European guidelines and regulations. The experiment was approved by the Auvergne regional ethics committee for animal experimentation, approval number CE1-10.

### Wethers, diets and treatments

Twelve 3-year old rumen-cannulated Texel wethers were used to examine the effect of bacterial probiotic supplementation on rumen microbial and fermentation characteristics during induced lactic acidosis and SARA. The wethers weighed 60.7 ± 3.3 kg (mean ± SD) at the start of the experiment and were housed in individual stalls (1.0 × 1.50 m) with feed-bunks and free access to water and mineralized salts blocks. The 12 wethers were allocated to three groups differing in the nature of the feed challenge (wheat, corn or beet pulp) used to induce acidosis. Within each group, the four wethers were randomly assigned to four treatments in a 4 × 4 Latin square design with 24-d periods. Treatments were: 1) control without probiotics (**C**), 2) *Propionibacterium* P63 (**P**), 3) *Lactobacillus plantarum* strain 115 plus P (**Lp + P**) and 4) *Lactobacillus rhamnosus* strain 32 plus P (**Lr + P**). Before their administration, the different treatments were prepared in gelatin capsules (2 g/d), and then introduced in the rumen through the cannula just before the morning feeding or acidosis induction, at a dose of 1 × 10^11^ CFU/wether/d. The wethers on treatment C received only the carrier composed of lactose. The probiotics were specially prepared for this study by Danisco SAS (Dangé-Saint-Romain, France).

In the first 21 d of each period (adaptation period), the wethers were fed at 90% of their *ad libitum* intake in two equal portions (0900 h and 1600 h) with a basal non-acidogenic diet made of alfalfa hay and wheat-based concentrate (4:1 ratio on dry matter basis). This was followed by three consecutive days of acidosis induction (feed challenge period) where the wethers were intraruminally dosed with rapidly fermentable carbohydrates [13]. Briefly, the morning feeding was replaced by an intraruminal supply of ground concentrate (3 mm screen) representing 1.2% of body weight (BW). Three types of concentrates differing in the nature and degradation rate of their carbohydrates were used: wheat (readily fermentable starch), corn (slowly fermentable starch) and beet pulp (easily digestible fibers) to induce lactic acidosis, butyric SARA and propionic SARA, respectively. At 1600 h the wethers received 520 g of hay to help them restore their ruminal buffering capacity. The chemical composition of the feeds used in the basal diet and feed challenges for acidosis induction is indicated in Table 1.

**Table 1:** Chemical composition of the feeds used in basal diet and in feed challenges for acidosis induction (g/100 g DM)

	**Basal diet**^**1**^		**Feed challenges**^**2**^		
	**Hay**	**Concentrate**^**3**^	**Wheat**	**Corn**	**Beet pulp**
NDF	68.1	8.2	17.7	15.4	38.9
ADF	40.7	4.9	4.3	3.3	19.9
Starch	nd^4^	65.6	62.0	72.4	nd
CP	7.3	14.3	14.1	8.8	8.6

^1^ Natural grassland hay:wheat-based concentrate (4:1 ratio on DM basis).

^2^ Feed challenges: 1.2% body weight (BW) of ground wheat, corn or beet pulp was intraruminally dosed each morning of the feed challenge period. BW was 60.7 ± 3.3 kg at the beginning of the experiment.

^3^ Concentrate: wheat based concentrate with 3% molasses.

^4^ nd: not detected.

To minimize the carryover from period to period and help the rumen return more rapidly to an equilibrium state (especially in the case of lactic acidosis), the rumen of each wether was manually half-emptied on the last day of periods 1 to 3, and replaced by a rumen content isolated from three additional wethers fed the same basal diet.

### Rumen sample collection and treatments before analysis

During the 3-d feed challenge period, ruminal content samples (200 g) were taken each day from the ruminal ventral sac 1 h before, and 3 h and 6 h after intraruminal feed dosing.

Ruminal pH was immediately measured with a portable pH-meter (CG840, electrode Ag/AgCl, Schott Geräte, Hofheim, Germany). The samples were then treated for measurement of microbial and fermentation characteristics as follows: on d1 and d3 at −1 h and 3 h relative to intraruminal dosing, 30 g of ruminal content was immediately taken to the laboratory for enzyme extraction from the solid-adherent microorganisms (SAM) under anaerobic conditions. At the same time, 30 g of ruminal content was homogenized in ice using a Polytron grinding mill (Kinematica GmbH, Steinhofhalde Switzerland) at speed 5, for two 1 min cycles with 1 min rest in ice between cycles. Two aliquots of 1.5 g were then stored at − 80°C until DNA extraction for bacterial qPCR and PCR-DGGE analysis. For each sampling time, an aliquot of ruminal contents was dried at 103°C for 24 h for dry matter (DM) determination. At all sampling times, 100 g of ruminal content was strained through a polyester monofilament fabric (250 μm mesh aperture) and the filtrate was used for analysis of volatile fatty acids (VFAs), lactate, NH_3_-N and for protozoa counting. For VFAs, 0.8 mL of ruminal filtrate was mixed with 0.5 mL of a 0.5 N HCl solution containing 0.2% (w/v) metaphosphoric acid and 0.4% (w/v) crotonic acid. For NH_3_-N, 5 mL of ruminal filtrate was mixed with 0.5 mL of 5% H_3_PO_4_. These samples were stored at − 20°C until analysis. For protozoa, 3 mL of the fresh filtrate was mixed with 3 mL of methyl green, formalin and saline solution (MFS) and preserved from light until counting.

### Measurements

#### Bacterial quantification by quantitative PCR

Genomic DNA was extracted using the FastDNA® Spin Kit, and purified with the GeneClean® Turbo Kit (MP Biomedicals, Illkirch, France) according to the manufacturer’s instructions with minor modifications. Briefly, 250 mg of frozen milled ruminal contents was weighed into the tube provided containing silica beads and lysis buffer. Bacteria were lyzed using a beadbeater (Precellys 24, Bertin Technology, France). The yield and purity of the extracted DNA were assessed by optical density measurement with a Nanoquant Infinite M200 spectrophotometer (Tecan Austria GmbH, Grödig, Austria), using a dedicated quantification plate. Absorbance intensity at 260 nm was used to assay nucleic acids in 2 μL of sample. Absorbance ratios 260/280 and 260/230 were used to check sample purity.

The quantitative PCR (qPCR) was carried out using the StepOnePlus^TM^ real-time PCR system and software (Applied Biosystems, Courtaboeuf, France). Detection was based on SYBR green chemistry. Total bacteria and selected species were quantified by targeting the *rrs* gene (Table 2). The reaction mix contained 0.75 × SYBR Premix Ex Taq (Lonza Verviers SPRL, Verviers, Belgium), 0.5 μM of each forward and reverse primer and 80 ng of DNA template. Each reaction was run in triplicate in a final volume of 20 μL in 96-well reaction plates (Applied Biosystems, Courtaboeuf, France). Amplification programs consisted of one cycle at 95°C (10 s) and 40 denaturing cycles at 95°C (15 s) and annealing at 60°C (30 s) for total bacteria, *Prevotella* genus, *Ruminococcus albus*, *Fibrobacter succinogenes* and *Ruminococcus flavefaciens*. For *Streptococcus bovis* the annealing temperature was 63.9°C (30 s), while the amplification of *Lactobacillus* consisted of one cycle at 95°C (10 min) and 40 denaturing cycles at 95°C (30 s) and annealing at 60°C (1 min). Absolute quantification was carried out for all bacteria using specific 16 S rDNA standards from *R. flavefaciens* c94 (ATCC 19208), *R. albus* 7(ATCC 27210), *F. succinogenes* S85 (ATCC 19169), *S. bovis* (DSM 20480), *P. bryantii* B14 (DSM 11371), and *Lb. acidophilus*. The results for counting of each species are expressed as % of total bacteria/g DM of rumen content. Only assays that fell in the range 90–110% of efficiency and with *r*^2^ ≥ 0.98 were considered for further analysis.

**Table 2:** ** *rrs* gene based primers used for qPCR quantification and PCR-DGGE**

**Target organism**	**Primer set**	**Primer sequences 5' - 3'**	**Assay**	**Reference**
Total bacteria	520 F	AGCAGCCGCGGTAAT	qPCR	[14]
	799 R2	CAGGGTATCTAATCCTGTT		
*Fibrobacter*	FibSuc3F	GCGGGTAGCAAACAGGAT TAGA	qPCR	[15]
*succinogenes*	FibSuc3R	CCCCCGGACACCCAGTAT		
*Ruminococcus*	RumAlb3F	TGTTAACAGAGGGAAGCAAAGCA	qPCR	[15]
*albus*	RumAlb3R	TGCAGCCTACAATCCGAACTAA		
*Ruminococcus*	RumFla3F	TGGCGGACGGGTGAGTAA	qPCR	[15]
*flavefaciens*	RumFla3R	TTACCATCCGTTTCCAGAAGC T		
*Genus*	PrevGen4F	GGTTCTGAGAGGAAGGTCCCC	qPCR	[15]
*Prevotella*	PrevGen4R	TCCTGCACGCTACTTGGCTG		
*Streptococcus*	StrBov2F	TTCCTAGAGATAGGAAGTTTCTTC GG	qPCR	[15]
*bovis*	StrBov2R	ATG ATG GCA ACT AAC AAT AGG GGT		
*Genus*	Lacto 05 F	AGC AGT AGG GAA TCT TCC A	qPCR	[16]
*Lactobacillus*	Lacto 04R	CGCCACTGGTGTTCYTCCATATA		
Total bacteria	**GC** + Eub340F	**CGCCCGCCGCGCGCGGCGGGCGGGGCG GGGGCACGGGGGG**TCCTACGGGAGGCAGCAG	DGGE	[17-19]
	HDA2R	GTA TTA CCG CGG CTG CTG GCA C		

#### PCR and Denaturing Gradient Gel Electrophoresis (DGGE)

The V3 region of the bacterial *rrs* gene was amplified in PCR using primers Eub340F [17,18] and HDA2R [19]. The Eub340F primer was modified for broader bacterial coverage and was tested in association with HDA2R on pure culture microorganisms. In all cases, the primer pair produced single PCR products that matched the target sequence from known microorganisms (E. Galbraith, unpublished data). For DGGE, a 40 bp GC clamp was added to the 5’ end of the forward primer Eub340F (Table 2). In 50 μL final volume, each reaction contained 2.5 units/μL of Platinum Taq DNA Polymerase (Invitrogen, Carlsbad, CA), 1 × PCR Buffer, 200 μM dNTP mix, 80 ng of template DNA, and 0.5 μM of each primer. PCR was performed using the GeneAmp PCR System 2700 thermocycler (Applied Biosystems, Foster City, CA). We used the PCR program described by Smith and Mackie [20] with the following modification: 20 touchdown cycles were used instead of 10, and the annealing temperature was decreased by 0.5°C every cycle (instead of 1°C) from 65 to 55°C. PCR amplification products were analyzed on a 1% E-gel 96 agarose (Invitrogen, Carlsbad, CA). Amplicon size and concentration were estimated using E-gel Low Range Quantitative DNA Ladder (Invitrogen, Carlsbad, CA) and Syngene Bioimaging System and GeneSnap software (Syngene, Frederick, MD).

The DGGE gels were cast using the DCode universal mutation detection system (BioRad, Hercules, CA) as previously described [19]. Briefly, polyacrylamide gels (8%) were prepared and run using 0.5 × TAE buffer. A gradient maker was used (CBS Scientific Co., Del Mar, CA) to prepare gels that contained a 30–60% gradient of urea and formamide increasing in the direction of electrophoresis. A 100% denaturing solution contained 40% (vol/vol) formamide and 7.0 M urea. The polyacrylamide gel wells were loaded with 10 μL of PCR product and 10 μL of 2 × loading dye (0.05% bromophenol blue, 0.05% xylene cyanol and 70% glycerol). Within each feed challenge group, the DNA samples were pooled by treatment after the PCR amplification, and then loaded on the gel to assess the global community structure. The electrophoresis was conducted with a constant voltage of 130 V at 55°C for about 4 h. Gels were stained with ethidium bromide solution (0.5 μg/mL, 10 min), and washed (0.5 × TAE buffer, 10 min). Gel images were acquired using Syngene Bioimaging System and GeneSnap software (Syngene, Frederick, MD). The GelCompar II v5.10 software (Applied Maths, Belgium) was used to analyze the DGGE gels. To normalize the differences among gels, the same standard was used for each gel. The percentage of similarity between gel standards was 96%.

The DGGE profiles were normalized and compared using hierarchical clustering to join similar profiles in groups [21]. To this end, all the images of DGGE gels were matched using the standard and the bands were quantified after a local background subtraction. A 1% tolerance in the band position was applied. The cluster analysis was based on Dice’s correlation index and the clustering was done with the unweighted pair-group method using arithmetic averages (UPGMA).

#### Protozoa counting

Protozoa were enumerated in a Dolfuss cell (Elvetec Services, Clermont-Ferrand, France), using a photonic microscope according to the method of Jouany and Senaud [22].

#### Polysaccharidase activities of solid-associated microorganisms

Polysaccharidase activities involved in the degradation of plant cell wall (EC 3.2.1.4 - cellulase and EC 3.2.1.8 - endo-1,4-β-xylanase) and starch (EC 3.2.1.1 - α-amylase) were determined from the solid-adherent microorganisms (SAM) as already described [23]. Briefly, 30 g of solid phase was washed with 350 mL anaerobic MES buffer (2-(*N*-morpholino) ethane sulfonic acid; pH 6.5, 39°C) to remove the non-associated and loosely-associated microbes, and then recovered by filtration (100 μm). A 5 g sample of washed digesta containing the SAM was cut in an anaerobic environment, suspended in 25 mL of anaerobic MES buffer and stored at −80°C pending enzyme extraction. The SAM fraction was broken up by defrosting and ultrasonic disintegration (four 30 s periods with 30 s intervals at 4°C; Branson 250 D 200 W, Elvetec services, Clermont-Ferrand, France). Samples were centrifuged (15,000 *g*, 15 min, 4°C) and the supernatant containing the released enzymes was stored in capped tubes at −80°C before assay. Polysaccharidase activities were determined by assaying the amount of reducing sugars released from purified substrates (Birchwood-xylan, Sigma X-0502; carboxymethylcellulose, Sigma C-5678; potato starch, Sigma S-2004) after incubation for 1 h at 39°C. Briefly, the reducing sugars were converted into colored products using PAHBAH (4-hydroxybenzhydrazide) in the presence of bismuth and quantified spectrophotometrically at 410 nm [24]. The protein content of the enzyme preparations was determined according to Pierce and Suelter [25] using bovine serum albumin as standard in 96-well plates using the Nanoquant Infinite M200 spectrophotometer (Tecan Austria GmbH, Grödig, Austria). Enzyme activities were expressed in μmol of reducing sugar released per g of DM per hour (total activity) and in μmol of reducing sugar released per mg protein per hour (specific activity).

#### Fermentation parameters

Volatile fatty acids and lactate concentrations were determined by gas chromatography (CP 9002 Gas Chromatograph, Chrompack, Middelburg, Germany) and an enzymatic method (Enzyplus EZA 891+, D/L-Lactic Acid, Raisio Diagnostics, Rome, Italy) respectively as described in Lettat et al. [13]. For NH_3_-N, thawed samples were centrifuged at 10,000 *g* for 10 min and NH_3_-N concentration was determined in the supernatant using the Berthelot reaction [26]. The reaction was carried out in duplicate in 96-well plates and read using the Nanoquant Infinite M200 spectrophotometer (Tecan Austria GmbH, Grödig, Austria).

### Statistical procedure

All the data were analyzed in repeated time using the MIXED procedure of SAS, with SP(POW) as covariance structure for unequally spaced data. Within each Latin square, the period (1 to 4), treatment (C *vs.* P, *vs.* Lp *+* P*, vs.* Lr + P), feed challenge day (d1 *vs.* d3) and time (−1 *vs.* + 6 h and −1 *vs.* + 3 h for rumen fermentation and microbiological parameters, respectively) were considered as fixed effects, and animal as random. Results were considered significant for *P* ≤ 0.05. When treatment was significant, means were separated using orthogonal contrasts: C *vs.* (P, Lp + P, Lr + P); P *vs.* (Lp + P, Lr + P) and Lp + P *vs.* Lr + P.

## Results and discussion

Lactic acidosis is characterized by a mean ruminal pH < 5.2 associated with high lactate concentration [27], whereas for SARA, where the condition is subtler, several definitions have been proposed [13,28,29]. For the purpose of this study, we used a mean value of 6.25 as the ruminal pH benchmark for SARA determination [30]. Based on the ruminal pH and fermentation patterns observed in this study during the 3-d feed challenge periods, acidosis induction was attained on d3 (data not shown). Lactic acidosis was induced with wheat, whereas butyric and propionic SARA were induced with corn and beet pulp, respectively. These results are similar to those of our previous study [13] in which these three acidosis forms were induced in wethers using the same feeds.

Irrespective of the acidosis, we also observed that the differences among treatments were accentuated during the three days of feed challenges, being maximal and significant only on the third day. Consequently, only data related to the effect of probiotic supplementations on the rumen characteristics on d3 are reported and discussed here.

### Lactic acidosis induced by wheat

**Figure 1:**
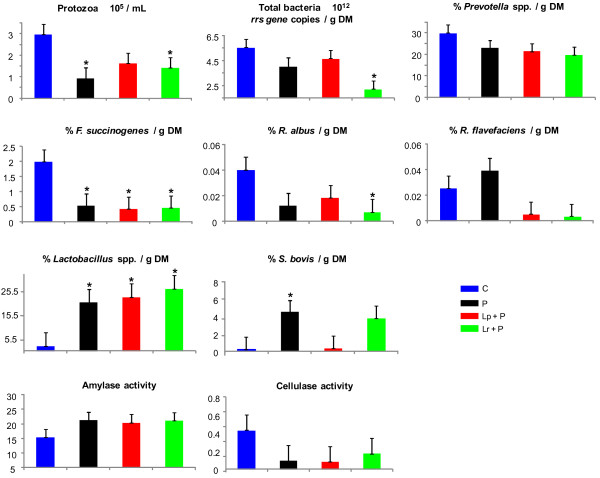
**Effects of bacterial probiotic supplementation on the rumen microbial parameters during wheat-induced lactic acidosis.** Acidosis was induced during 3 consecutive days. Protozoa, bacteria and polysaccharidase activities were quantified 3 h after acidosis induction on day 3. Bacterial species are expressed as % of total bacteria per gram of dry matter (DM). Polysaccharidase activities are expressed as μmol of reducing sugar/mg protein/h. The treatments were identified as C = control without probiotic; P = *Propionibacterium* P63; Lp + P = *L. plantarum* + P63; Lr + P = *L. rhamnosus* + P63. Each single point is a mean of 4 data points from the 4-periods Latin square. Error bars represent standard error of the means. Probiotic treatments that significantly differ from control are indicated by * for *P* ≤ 0.05.

Lactic acidosis is a rare accidental pathology in which the ruminal ecosystem is completely disturbed. In this experiment, the mean and minimum ruminal pH were 5.25 and 4.86 respectively, concentration of lactate reaching ~ 34 mM and that of total VFAs 94 mM for control wethers (Table 3). These values are classically observed in lactic acidosis situations [13,31]. Compared with the control animals, a drastic decrease in total bacteria was observed for Lr + P fed wethers (*P* < 0.05; Figure 1), whereas feeding P and Lr + P decreased the population of protozoa (*P* < 0.05). Without significantly affecting fibrolytic activities (cellulase and xylanase), the three probiotic treatments reduced the proportion of the cellulolytic bacterium *F. succinogenes*, Lr + P decreased *R. albus* while *R. flavefaciens* was not affected. The growth of lactate-producing bacteria (*Lactobacillus* spp. and *S. bovis*) was enhanced by probiotic supplementation. *S. bovis* proportion was highest for P-fed wethers whereas *Lactobacillus* spp. became a predominant bacterial group: from 1.7% in C up to 25% of total bacteria in probiotic-supplemented wethers (*P* < 0.05). Specific amylase activity was not significantly affected by probiotic supplementation, but the total activity was increased in P-fed wethers (*P* < 0.05; data not shown). As expected, lactobacilli proliferation caused an increase in lactate concentration that reached more than 60 mM in probiotic-fed wethers (*P* < 0.05; Table 3), whereas total VFA concentrations were less than 35 mM for P and Lr + P (*P* < 0.05), suggesting a decrease in microbial fermentative activity and a shift towards lactate production at the expense of VFAs (*P* < 0.05). It could be argued that the increase was due to the addition of exogenous lactobacilli. However, wethers that received only *Propionibacterium* P63 exhibited similar proportions of *Lactobacillus* spp. to those supplemented with a combination of *Propionibacterium* and *Lactobacillus* (*P* = 0.5). Therefore, it seems that the lactobacilli quantified were indeed autochthonous symbionts and that *Propionibacterium* P63 may improve the growth of this bacterial group. Lactate accumulation in the rumen can be explained by the increase in lactate producers as discussed above, but it might also be coupled to a decreased number or activity of lactate-utilizers. The bacterium *M. elsdenii*, which is considered to be the most efficient lactate-utilizer [10,32], was not detected in our samples (data not shown). As a result, lactate accumulation induced a drop in mean and minimum ruminal pH, compared with C wethers (−0.70 and −0.33 pH units on average; *P* < 0.05). Among probiotic treatments, pH was lowest for Lr + P, intermediate for P and highest for Lp + P (*P* < 0.05). P and Lr + P decreased propionate and butyrate proportions, whereas minor VFAs were reduced by all three probiotics (*P* < 0.05). The concentration of NH_3_-N was reduced for P and Lr + P fed wethers (*P* < 0.05), whereas it was numerically lower for those fed Lp + P. This decrease in NH_3_-N may be due to a decrease in deamination activity, as the proportion of *Prevotella* spp., a dominant bacterial genus that plays a central role in amino acid deamination in the rumen [33], was numerically lower in wethers fed with Lp + P and Lr + P (*P* = 0.1 and 0.06; respectively). In addition, probiotic supplementation increased ethanol concentration, a minor fermentation product that does not accumulate in the rumen except during lactic acidosis [34,35] because of the heterofermentative metabolism of glucose by lactobacilli, which leads to lactate and ethanol production [36].

**Table 3:** Effects of bacterial probiotic supplementation on rumen fermentation characteristics during acidosis induced by feed challenges

	**Treatments**^**1**^					** *P* value (Prob *vs.* C)**^**2**^		
	**C (n = 4)**	** *P* (n = 4)**	** *Lp + P* (n = 4)**	** *Lr + P* (n = 4)**	**SEM**	** *P* **	** *Lp + P* **	** *Lr + P* **
*Wheat-induced lactic acidosis*								
Ruminal pH								
Mean	5.25	4.55	4.76	4.33	0.15	0.001	0.02	0.0001
Minimum	4.87	4.28	4.45	4.17	0.19	0.03	0.12	0.01
Total VFAs, mM	93.6	33.9	76.7	33.5	14.4	0.01	0.32	0.001
Acetate^3^, mol %	72.6	87.0	78.1	92.5	4.10	0.01	0.34	0.001
Propionate, mol %	12.2	6.63	10.6	3.82	2.49	0.10	0.63	0.02
Butyrate, mol %	12.8	5.79	10.2	3.52	1.94	0.01	0.33	0.001
Minor VFAs^4^, mol %	2.33	0.56	1.11	0.14	0.40	0.001	0.02	0.0001
Lactate, mM	33.8	71.1	64.9	79.6	9.28	0.005	0.02	0.001
NH_3_-N, mM	6.53	3.58	4.25	2.44	1.16	0.03	0.09	0.003
Ethanol, mM	6.57	12.4	17.2	14.4	1.85	0.02	0.0001	0.003
*Corn-induced butyric subacute acidosis*								
Ruminal pH								
Mean	5.49	5.61	5.74	5.65	0.08	0.30	0.03	0.18
Minimum	5.17	5.28	5.63	5.46	0.12	0.50	0.01	0.09
Total VFAs, mM	107	85.7	81.6	94.4	7.79	0.03	0.01	0.19
Acetate, mol %	63.2	67.4	68.7	66.9	1.75	0.08	0.03	0.13
Propionate, mol %	17.0	14.2	14.5	15.5	1.09	0.07	0.19	0.31
Butyrate, mol %	16.9	14.7	12.1	13.5	1.41	0.26	0.02	0.09
Minor VFAs, mol %	2.88	3.68	4.29	4.09	0.44	0.20	0.02	0.05
Lactate, mM	3.40	3.78	3.22	3.49	0.86	0.71	0.87	0.92
NH_3_-N, mM	0.74	0.73	0.71	1.15	0.98	0.99	0.98	0.76
Ethanol, mM	3.15	3.60	2.72	2.74	0.36	0.38	0.40	0.42
*Beet pulp-induced propionic subacute acidosis*								
Ruminal pH								
Mean	5.67	5.94	5.87	5.93	0.08	0.02	0.08	0.02
Minimum	5.55	5.84	5.72	5.83	0.11	0.05	0.27	0.06
Total VFAs, mM	114	112	104	100	6.66	0.89	0.33	0.16
Acetate, mol %	67.4	68.6	68.4	67.8	1.15	0.46	0.55	0.79
Propionate, mol %	22.5	21.5	21.9	22.3	0.83	0.38	0.61	0.88
Butyrate, mol %	8.52	8.40	8.18	8.34	0.49	0.86	0.85	0.77
Minor VFAs, mol %	1.50	1.48	1.52	1.46	0.26	0.94	0.96	0.91
Lactate, mM	2.71	2.01	1.52	2.01	1.46	0.73	0.56	0.73
NH_3_-N, mM	0.55	0.51	0.57	0.57	0.74	0.97	0.99	0.98
Ethanol, mM	3.34	3.22	2.64	2.84	0.48	0.86	0.31	0.47

^1^ Treatment with C = control without probiotic; P = *Propionibacterium* P63; Lp + P = *L. plantarum* + P63; Lr + P = *L. rhamnosus* + P63. ^2^ Effect of each probiotic treatment *vs.* control wether (C). ^3^ Individual VFAs are expressed in % of total VFAs. ^4^ Minor VFAs: sum of iso-butyrate, iso-valerate, valerate and caproate. The fermentation characteristics were determined on d3 at 6 h after feed challenges induced acidosis.

According to the fermentation and microbial characteristics, the negative effects induced by probiotic supplementation were more marked for P and Lr *+* P than for Lp + P. A possible explanation for this difference could be that the proportion of *S. bovis* was higher in wethers treated with P (*P* < 0.05) and almost reached significance for Lr *+* P-fed wethers (*P* = 0.06) as compared with those supplemented with Lp + P (*P* = 0.9). Thus *S. bovis* could be considered as a worsening factor rather than an initial cause of the chain of events resulting in lactic acidosis in ruminants [37-39]. Also, in contrast to P and Lr + P feeding, the supplementation with Lp + P did not reduce the protozoa population (*P* = 0.16). Thus maintaining a higher protozoal population, which is known to stabilize rumen pH, may explain why Lp + P was the “least bad” of the three probiotic treatments tested [4,40].

**Figure 2:**
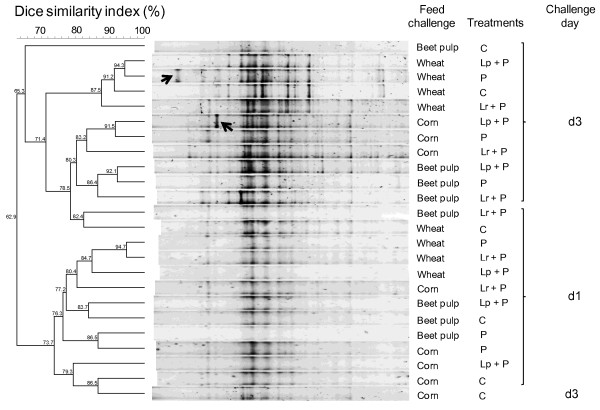
**Effect of acidosis induction and bacterial probiotic supplementation on rumen bacterial diversity.** DGGE profiles of PCR-amplified *rrs* gene fragments of bacterial communities from the rumen of sheep before (d1 at −1 h) and the last day (d3 at 3 h) of wheat-induced lactic acidosis, corn-induced butyric or beet-pulp propionic subacute acidosis. Each sample is a pool of 4 wethers (from the 4-period Latin square) within the same treatment with C = control without probiotic; P = *Propionibacterium* P63; Lp + P = *L. plantarum* + P63; Lr + P = *L. rhamnosus* + P63. The cluster analysis was based on Dice’s correlation index and the unweighted pair-group method with arithmetic averages (UPGMA). Arrows indicate a specific band for P during lactic acidosis and another one for Lp + P during butyric subacute acidosis.

The DGGE analysis of the ruminal bacterial population showed that regardless of the feed used, most of the d1 and d3 samples clustered in two different groups, with 73.7 and 65.3% similarity, respectively (Figure 2). Separation into distinct groups indicates that the bacterial structure was modified by acidosis induction. On d3, DGGE profiles from wethers challenged with wheat clustered together (87.5% similarity). The number of bands, interpreted as an index of richness, was greater on d3 than on d1, with an average of 35 *vs.* 22 bands, respectively. This result is somewhat surprising because lactic acidosis is thought to induce a less rich bacterial community owing to the large increase in lactobacilli and decrease in other bacteria as revealed by qPCR [41]. The higher richness could be due to an increased diversity of lactate-producing bacteria. In future studies, the diversity of lactobacilli and streptococci species and strains should be assessed by the use of second generation sequencing methods or specific techniques such as ribotyping. Unfortunately, explanations are still lacking due to the absence of similar studies in the literature. In addition, a band only present at d3 for wethers supplemented with P has been detected. Further identification of this specific band together with other bands that appeared or disappeared following lactic acidosis induction will enhance our knowledge on how the bacterial communities are affected by acidosis onset and probiotic supplementation.

In these experimental conditions, the probiotics used were not effective in alleviating the onset of rumen lactic acidosis in challenged wethers. Instead, supplementation with probiotics had a worsening, catalytic effect on lactic acidosis by enhancing lactate-producing bacteria proliferation and altering fermentation parameters (decrease in pH and VFAs, increase in lactate concentration), important for the development of this digestive disorder [4,42]. In conclusion, bacterial probiotics such as those of the type tested in this work cannot be used to prevent lactic acidosis onset in ruminants. Good dietary management practices are still the best way to avoid this rare accidental digestive disorder.

#### Butyric and propionic SARA induced by corn and beet pulp

**Figure 3:**
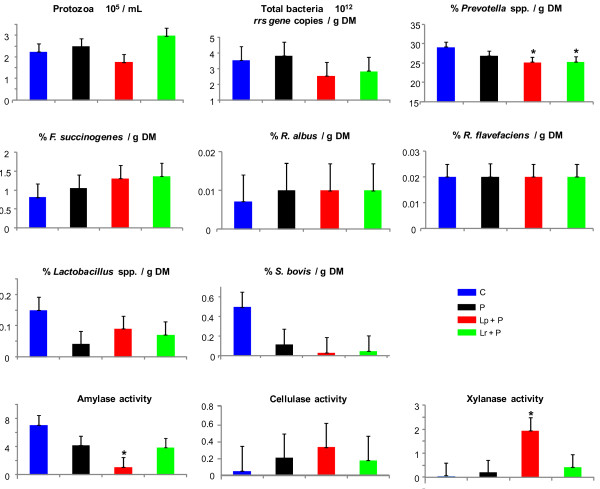
**Effects of bacterial probiotic supplementation on the rumen microbial parameters during corn-induced butyric subacute acidosis.** Acidosis was induced during 3 consecutive days. Protozoa, bacteria and polysaccharidase activities were quantified 3 h after acidosis induction on day 3. Bacterial species are expressed as % of total bacteria per gram of dry matter (DM). Polysaccharidase activities are expressed as μmol of reducing sugar/mg protein/h. The treatments were identified as C = control without probiotic; P = *Propionibacterium* P63; Lp + P = *L. plantarum* + P63; Lr + P = *L. rhamnosus* + P63. Each single point is a mean of 4 data points from the 4-period Latin square. Error bars represent standard error of the means. Probiotic treatments that significantly differ from control are indicated by * for *P* ≤ 0.05.

In C wethers, butyric acidosis induced by corn challenge was characterized by a mean ruminal pH of 5.49, total VFA concentration of ~ 107 mM with ~ 17% of butyrate and a weak concentration of lactate (3.4 mM; Table 3), in agreement with previous reports of butyric SARA [13,40,43]. Regarding the microbial composition and activities (Figure 3), total and cellulolytic bacteria and protozoa were not affected by probiotic supplementation. Feeding Lp + P and Lr + P resulted in lower *S. bovis* and *Prevotella* spp. proportion (*P* < 0.05), while the decrease in *Lactobacillus* spp. proportion almost reached significance in P-fed wethers (*P* = 0.06). The treatment Lp + P reduced both total (data not shown) and specific amylase activities and increased specific xylanase activity (*P* < 0.05), whereas specific amylase activity was numerically lower in wethers fed with Lr + P. These moderate microbiological shifts were accompanied by some changes in the fermentation patterns. Wethers supplemented with Lp *+* P and Lr *+* P had a higher pH nadir compared with C (+ 0.46 pH units; *P* < 0.05), but only Lp + P had higher mean ruminal pH (+ 0.25 pH units, *P* < 0.05). The rise in pH was associated with a decrease in total VFA concentration (− 24%, *P* < 0.05), and butyrate proportion (*P* < 0.05) and an increase in acetate and minor VFAs (*P* < 0.05). Feeding P also reduced total VFAs (*P* < 0.05), and numerically changed individual VFAs proportions as did Lr + P. However, neither probiotic significantly affected mean ruminal pH.

**Figure 4:**
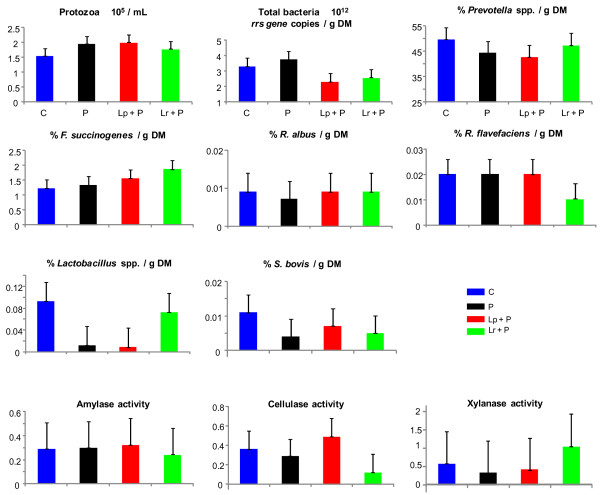
**Effects of bacterial probiotic supplementation on the rumen microbial parameters during beet pulp-induced propionic subacute acidosis.** Acidosis was induced during 3 consecutive days. Protozoa, bacteria and polysaccharidase activities were quantified 3 h after acidosis induction on day 3. Bacterial species are expressed as % of total bacteria per gram of dry matter (DM). Polysaccharidase activities are expressed as μmol of reducing sugar/mg protein/h. The treatments were identified as C = control without probiotic; P = *Propionibacterium* P63; Lp + P = *L. plantarum* + P63; Lr + P = *L. rhamnosus* + P63. Each single point is a mean of 4 data points from the 4-period Latin square. Error bars represent standard error of the means. Probiotic treatments that significantly differ from control are indicated by * for *P* ≤ 0.05.

Propionic SARA was characterized in C wethers by a mean ruminal pH of 5.67, total VFA concentration of 114 mM, 22.5% of propionate and less than 3 mM of lactate (Table 3). These findings are in agreement with earlier reported studies on propionic SARA induced by intraruminal dosing of beet pulp [13] and in normally fed cattle [44,45]. Probiotic supplementation did not affect significantly the microbial composition, polysaccharidase activities and fermentation patterns that remained similar among treatments (Figure 4). For amylase activity, this could be explained by the fact that beet pulp does not contain starch but sucrose, and that the development of amylase activity requires starch availability [46]. Without clear effects on microbial and fermentation patterns, explanations are still lacking on how the probiotics increased mean (+ 0.27 pH units on average, for P and Lr + P) and minimum ruminal pH (0.29 pH units on average, for P and Lr + P). In contrast to qPCR, which showed subtle changes in the bacterial community, DGGE analysis revealed that bacterial structure was affected by probiotic supplementation, insofar as supplemented wethers clustered together with 83.2 and 86.4% similarity for butyric and propionic SARA, respectively (Figure 2). These complementary results indicate that shifts in the bacterial communities may result in unchanged fermentation patterns and that these shifts concerned bacterial groups that differ from those targeted by qPCR. Also, similarly to lactic acidosis, the richness index was greater at d3 than at d1, with an average of 26 *vs.* 18 and 27 *vs.* 22 bands for butyric and propionic SARA, respectively. This result conflicts with recent work reporting a decrease in bacterial richness when SARA was induced in dairy cows [2]. This discordance could be due to the mode of acidosis induction (intraruminal dosing *vs.* normal feeding) or the nature of the samples, as DNA extraction was achieved from ruminal liquid in the reported study, whereas we used whole ruminal content (liquid + solid). Also, wethers supplemented with probiotics exhibited a higher richness index than controls, with 31 *vs.* 21 and 31 *vs.* 23 bands on average for butyric and propionic SARA, respectively. For butyric SARA, an intense band was observed with Lp + P. Sequencing and identification of the band can establish a causal link between a species and changes observed in pH and xylanase activity. As for lactic acidosis, further sequencing experiments are required to enhance our knowledge of how SARA and probiotics affect the rumen bacterial structure and activity.

Among the few studies published on the use of bacterial probiotics, only two [47,48] tested the effects of *Lactobacillus* and *Propionibacterium* strains on ruminal fermentation during SARA. One of the studies tested *P. acidipropionici* P15 alone (P15; 1 × 10^9^ CFU/d) or in combination with *E. faecium* 212 (PE; 1 × 10^9^ + 1 × 10^9^ CFU/d) on steers fed a 90% steam-rolled barley based diet. The probiotics did not affect ruminal pH, but P15 supplementation increased butyrate proportion and protozoa population with a concomitant reduction in amylolytic bacteria and *S. bovis* counts [47]. In the other study, *P. freudenreichii* PF24 in association with *Lb. acidophilus* 747 (1 × 10^9^ + 2 × 10^9^ CFU/d) or *Lb. acidophilus* 747 and *Lb. acidophilus* 45 (1 × 10^9^ + 2 × 10^9^ + 5 × 10^8^ CFU/d) given to mid-lactation Holstein dairy cows fed a 41% concentrate based diet did not affect the ruminal fermentations or pH, which was approximately 6.15 for control and probiotic-supplemented cows [48]. According to our present hypothesis that probiotics become effective when the ruminal ecosystem is unstable, it appears that the conditions were not acidotic enough in the study of Raeth-Knight et al. [48], whereas the effects reported by Ghorbani et al. [47] may indicate a decrease in acidosis risk even though the ruminal pH was not affected by probiotic supplementation [47]. In other studies reporting the use of probiotic bacteria, beneficial effects on ruminal pH were only observed for treatments associating bacteria and yeast [11,12], and never for bacteria alone [29,47-50]. Thus the beneficial effects on pH reported by Nocek et al. [11] and Chiquette [12] were probably not specific to the bacteria used, and may be attributed to *S. cerevisiae*, which has been shown to stabilize ruminal pH [8,9,51]. However, a synergistic effect cannot be excluded as, to our knowledge, there have been no studies comparing yeast and bacteria used alone and in association. The present work is the first to report a specific positive effect of bacterial probiotics on ruminal pH during SARA. The mode of action of these probiotics, consisting of *Lactobacillus* and *Propionibacterium* selected strains, could not be clearly associated with quantitative characteristics of the rumen microbial ecosystem such as bacterial and protozoal populations.

## Conclusion

This study shows for the first time that *Lactobacillus* and *Propionibacterium* probiotic strains may be effective in stabilizing ruminal pH and therefore preventing SARA risk, but they were not effective against lactic acidosis. The present results also suggest that the effectiveness of probiotics is compromised by ruminal fermentations, and are effective when the ruminal ecosystem is unstable. Although their mode of action needs to be further elucidated, we hypothesize that the effect of the probiotic strains used on ruminal pH was achieved by modulating the rumen microbiota, which was more diverse, by improving cellulolytic activity and by limiting the proliferation of lactic acid-producing bacteria. The combination of lactobacilli and *Propionibacterium* P63 seems to be more efficient in preventing SARA than P63 alone, possibly due to a synergistic effect between the strains.

## Abbreviations

BW, Body weight; CFU, Colony forming unit; DGGE, Denaturing gradient gel electrophoresis; DM, Dry matter; Lp + P, Lactobacillus plantarum plus P63; Lr + P, Lactobacillus rhamnosus plus P63; P63, Propionibacterium strain P63; qPCR, Quantitative PCR; SARA, Subacute ruminal acidosis; VFA, Volatile fatty acids.

## Competing interest

The probiotics used are the property of Danisco SAS.

## Author’s contribution

AL, PN, CM, MS, DPM and CB designed the study. CB initiated the funding from Danisco. AL, PN, CM, MS and DPM participated in the animal experiment. AL did the biochemical and molecular experiments, analyzed the data and drafted the manuscript. AL, PN, CM, DPM and CB revised the manuscript. All authors read and approved the final manuscript.
